# Evidence for phosphate-dependent control of symbiont cell division in the model anemone *Exaiptasia diaphana*

**DOI:** 10.1128/mbio.01059-24

**Published:** 2024-08-06

**Authors:** Nathan G. Faulstich, Alexis R. Deloach, Ykok B. Ksor, Gabriel H. Mesa, Daiven S. Sharma, Sebastian L. Sisk, Geoffrey C. Mitchell

**Affiliations:** 1 Wofford College, Spartanburg, South Carolina, USA; University of Hawaii at Manoa, Honolulu, Hawaii, USA; Oregon State University, Corvallis, Oregon, USA

**Keywords:** Symbiodiniaceae, *Aiptasia*, cell cycle, phosphate

## Abstract

Reef-building corals depend on symbiosis with photosynthetic algae that reside within their cells. As important as this relationship is for maintaining healthy reefs, it is strikingly delicate. When ocean temperatures briefly exceed the average summer maximum, corals can bleach, losing their endosymbionts. Although the mechanisms governing bleaching are unknown, studies implicate uncoupling of coral and algal cell divisions at high temperatures. Still, little is known regarding the coordination of host and algal cell divisions. Control of nutrient exchange is one likely mechanism. Both nitrogen and phosphate are necessary for dividing cells, and although nitrogen enrichment is known to increase symbiont density in the host, the consequences of phosphate enrichment are poorly understood. Here, we examined the effects of phosphate depletion on symbiont growth in culture and compared the physiology of phosphate-starved symbionts in culture to symbionts that were freshly isolated from a host. We found that available phosphate is as low in freshly isolated symbionts as it is in phosphate-starved cultures. Furthermore, RNAseq revealed that phosphate-limited and freshly isolated symbionts have similar patterns of gene expression for phosphate-dependent genes, most notably upregulation of phosphatases, which is consistent with phosphate recycling. Similarly, lipid profiling revealed a substantial decrease in phospholipid abundance in both phosphate-starved cultures and freshly isolated symbionts. These findings are important because they suggest that limited access to phosphate controls algal cell divisions within a host.

**IMPORTANCE:**

The corals responsible for building tropical reefs are disappearing at an alarming rate as elevated sea temperatures cause them to bleach and lose the algal symbionts they rely on. Without these symbionts, corals are unable to harvest energy from sunlight and, therefore, struggle to thrive or even survive in the nutrient-poor waters of the tropics. To devise solutions to address the threat to coral reefs, it is necessary to understand the cellular events underpinning the bleaching process. One model for bleaching proposes that heat stress impairs algal photosynthesis and transfer of sugar to the host. Consequently, the host’s demands for nitrogen decrease, increasing nitrogen availability to the symbionts, which leads to an increase in algal proliferation that overwhelms the host. Our work suggests that phosphate may play a similar role to nitrogen in this feedback loop.

## INTRODUCTION

Reef-building corals depend on energy produced by photosynthetic dinoflagellates (family Symbiodiniaceae) that live within their gastroderm. In return, heterotrophic corals provide these endosymbiotic algae with nitrogen (N) and phosphorus (P) (1). This symbiosis, however, is sensitive to anthropogenic challenges including rising sea surface temperatures, ocean acidification, and agricultural runoff (2). When stressed, corals bleach, losing or expelling their endosymbionts. Many bleached corals will simply die and others will fail to reproduce (3). Although marginal recovery is possible, it can take many years—time that will not be available as bleaching events become more frequent (4). As average sea surface temperatures continue to rise, more coral reefs around the world will be irrevocably altered.

Long-term maintenance of coral-algal symbiosis depends on coordination of cellular processes, including division. If coral cells proliferate without a corresponding increase in the algal population, then the benefits of symbiosis will be diluted. Likewise, if algal cell division outpaces coral cell division, it is possible that symbionts will drain hosts of vital nutrients or overburden them with production of reactive oxygen species. Strikingly, preferential expulsion of dividing algae has been demonstrated (5), suggesting a link between bleaching and failure of the coral host to maintain control of algal cell divisions at high temperatures.

Once symbiosis is established, it is unclear how coral and algal cell divisions are coordinated. Post-mitotic mechanisms have been suggested (6, 7); however, a growing body of evidence indicates that limited exchange of nutrients, particularly N, keeps symbiont populations in check (8–13). At the same time, there is compelling evidence that symbionts are P-limited as well. Both N and P are indispensable for dividing cells, allowing for biosynthesis of compounds such as amino acids, nucleic acids, and phospholipids. Although N enrichment is known to increase symbiont density *in hospite* (14, 15), as well as speed Symbiodiniaceae proliferation in culture (16), the consequences of P enrichment are not as well established. In two free-living dinoflagellates, the lack of phosphate initiates cell cycle arrest (17, 18). In Symbiodiniaceae, limited phosphate leads to upregulation of phosphatases (19). These enzymes free inorganic phosphate (P_i_) from phosphoesters; P_i_ can then be taken up by the symbiont through carrier proteins such as sodium-phosphate symporters (20). In corals, a meta-analysis of 15 studies found that enrichment with P alone has no impact on symbiont density but can work synergistically with N to enhance algal cell division (14). Thus, the authors concluded that P only limits Symbiodiniaceae growth *in hospite* when there is excess N, which does not appear to be the case (8, 11–13). Still, supporting the notion that Symbiodiniaceae are phosphate-limited *in hospite*, phosphatase activity is higher in freshly isolated symbionts than in symbionts in culture (19, 21).

This uncertainty led us to investigate the role of P in regulating symbionts *in hospite*. Our hypothesis is that Symbiodiniaceae are phosphate-limited within their cnidarian hosts, which hinders their proliferation. For this study, we used the model anemone *Exaiptasia diaphana*; this is a widely used model organism for studying many aspects of coral biology, including bleaching (22–24). Like its coral cousins, *Exaiptasia* hosts symbionts from the family *Symbiodiniaceae*, including the well-characterized *Breviolum minutum*. For laboratory studies, it has several advantages (25). For example, although *Exaiptasia* can reproduce sexually or asexually, its rapid asexual reproduction has led to the development of several clonal anemone lines that are shared among researchers (26, 27).

## RESULTS

### Sustained population growth of *B. minutum* in culture depends on P availability

**Fig 1:**
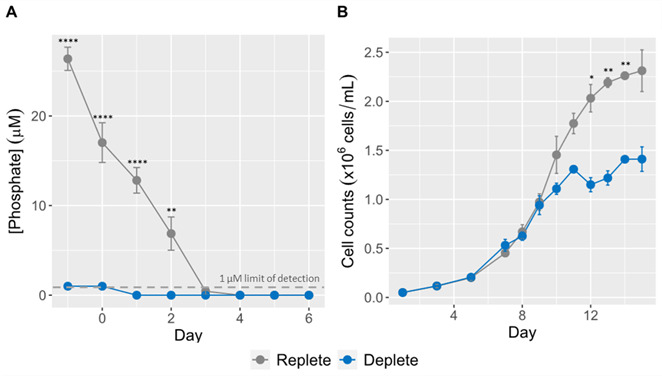
Sustained population growth of *B. minutum* in culture depends on phosphate availability. Cultures of *B. minutum* were seeded at 50,000 cells/mL in f/2 media containing either 36.2 µM (replete) or 10 µM (deplete) NaH_2_PO_4_. (**A**) The concentration of available phosphate in each culture was measured daily and plotted as the mean ± SD of six replicate cultures. A two-way ANOVA with Bonferroni multiple comparisons test was used to determine significant differences between the replete and deplete cultures (**P < 0.005, ****P < 0.0005). The experiment began (day 1) when phosphate became undetectable (<1 µM) in the deplete culture. (**B**) Starting on day 1, the density of each culture was determined by hemocytometer every 1–2 days and was plotted as the mean ± SD of six replicate cultures from two independent trials. A two-way ANOVA with Bonferroni multiple comparisons test was used to determine significant differences between the replete and deplete cultures (*P < 0.05, **P < 0.005).

To test the hypothesis that symbiont proliferation is limited by phosphate availability *in hospite*, we quantified the effects of phosphate limitation on *B. minutum* population growth in culture. *B. minutum* was seeded in f/2 made with 36.2 µM NaH_2_PO_4_ (replete) or 10 µM NaH_2_PO_4_ (deplete). The concentration of available phosphate in each culture was assessed daily until it became undetectable (<1 µM) in deplete samples (Fig. 1A). At this timepoint, referred to as day 1, the concentration of available phosphate in replete samples was ~13 µM.

Beginning on day 1, cultures were counted every 1–2 days. Growth curves for replete and deplete were at first indistinguishable, reaching exponential growth around day 7 (Fig. 1B). By day 10, deplete cultures began to plateau, reaching a maximal density of ~1.4 × 10^6^ cells/mL. Replete cultures continued expanding until day 12 when they too started to plateau, reaching a maximal density of ~2.3 × 10^6^ cells/mL. Notably, replete cultures, which were seeded with >3.5× the amount of phosphate, reached a maximal density <2× the maximal density of deplete cultures, suggesting that replete cultures may be ultimately limited by another resource (e.g., N or access to light). Furthermore, both deplete and replete cultures followed the same growth curve until day 10, which demonstrates that expansion of *B. minutum* cultures is limited at concentrations of phosphate much lower than the 1 µM detection limit of our assay. Thus, for subsequent experiments, day 10 samples were used because it is between days 10 and 12 that differences between the two conditions should be most obvious.

### Available phosphate is as low in freshly isolated *B. minutum* as in P-deplete cultures

**Fig 2:**
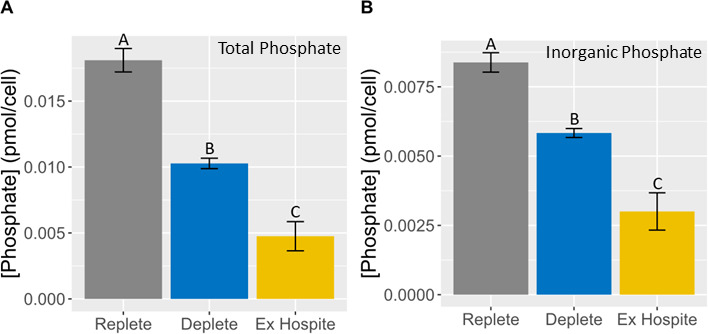
Available phosphate is lower in symbionts isolated from CC7-SSB01 anemones than in phosphate-depleted *B. minutum* in culture. When the effect of phosphate deprivation on population growth became apparent on day 10, *B. minutum* was collected from replete and deplete cultures and CC7-SSB01 anemones. The amount of total phosphate (**A**) and P_i_ (**B**) per symbiont was determined using a colorimetric assay and was plotted as the mean ± SD of three replicates. Groups that do not share a connecting letter are significantly different (one-way ANOVA followed by Tukey’s HSD, *P* < 0.005).

To determine whether symbionts are phosphate-limited *in hospite*, *B. minutum* was freshly isolated from two clonal lines of *Exaiptasia* (CC7-SSB01 and H2-SSB01), as well as from day 10 replete and deplete cultures. The amounts of total phosphate and P_i_ per symbiont were determined. Total phosphate per cell was significantly higher in replete than in freshly isolated symbionts (Fig. 2A; Fig. S1A) despite anemone feeding 3×/week *ad libitum*. In fact, total phosphate in freshly isolated symbionts was lower than in deplete, which were no longer growing at day 10. A similar trend was seen for P_i_ (Fig. 2B; Fig. S1B) except that the amount of P_i_ in each sample accounted for only ~50% of total phosphate.

### Phosphate-dependent gene expression is similar between freshly isolated *B. minutum* and phosphate-depleted cultures

**Fig 3:**
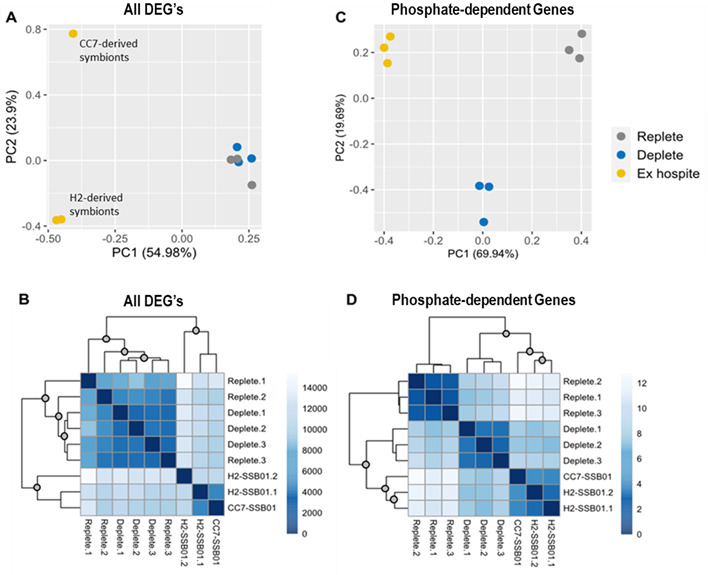
The expression pattern of phosphate-dependent genes is similar between freshly isolated and phosphate-depleted *B. minutum* in culture. When the effect of phosphate deprivation on population growth became apparent on day 10, *B. minutum* was collected from each culture and from two to three pooled *Exaiptasia*, and RNA was extracted. RNA sequencing was run and analyzed using a custom Galaxy pipeline. VST-normalized expression data were used for PCA (**A**) and to calculate sample-to-sample Manhattan distance (**B**). When replete and deplete cultures were compared, 47 DEGs were identified. VST-normalized expression data for this subset of DEGs was used for PCA (**C**) and to calculate sample-to-sample Manhattan distance (**D**). Statistical significance of hierarchical clustering was assessed using the sigclust2 R package (30). Gray circles on the nodes indicate statistical significance (*P* < 5 × 10^–5^).

Because phosphate availability in symbionts is similar in freshly isolated symbionts and phosphate-depleted cultures, RNAseq was performed to see if they have similar gene expression. Based on other studies (28), it is unsurprising that the highest number of differentially expressed genes (DEGs) was found between freshly isolated symbionts and those from culture (Data Sets S1 and S2). When comparing freshly isolated to replete, 5,703 genes were significantly upregulated and 5,126 were significantly downregulated. Likewise, freshly isolated compared to deplete had 5,667 genes that were significantly upregulated and 5,237 genes that were significantly downregulated. Principal component analysis (PCA) of normalized read counts revealed one principal component (PC1), which separates freshly isolated samples from cultured samples (Fig. 3A). Another principal component (PC2) separates the symbionts isolated from CC7-SSB01 from those isolated from H2-SSB01. This distance may be attributed to host strain; although both belong to the same species, they were collected from different environments and their distinctiveness is well documented (29). This distinction between CC7-derived and H2-derived symbionts is not apparent when examining sample-to-sample Manhattan distance of the same expression data (Fig. 3B). Instead, freshly isolated samples cluster separately from the cultures as expected with no further delineation among those groups.

To look specifically at genes responding to phosphate availability, replete and deplete samples were compared. In contrast to the >10,000 DEGs identified between freshly isolated and cultured symbionts, only 47 genes were differentially regulated between these samples (Data Set S3). Of these, 29 were significantly upregulated when phosphate was available (replete) and 18 were significantly downregulated. PCA using normalized read counts for these 47 phosphate-dependent genes yielded different results than the entire data set (Fig. 3C). Instead of separating just freshly isolated samples, PC1 clearly delineates all three groups. PC2 separates deplete from the other two groups, which implies that for some of these, freshly isolated and replete symbionts have similar expression. Overall, however, sample-to-sample Manhattan distance of normalized read counts for all 47 genes reveals significant clustering between freshly isolated and deplete symbionts, which are distinct from replete symbionts (Fig. 3D; Fig. S2). Although the distinction between CC7-derived and H2-derived symbionts is apparent here as it was in Figure 3A, the three freshly isolated samples form a significant cluster.

**Fig 4:**
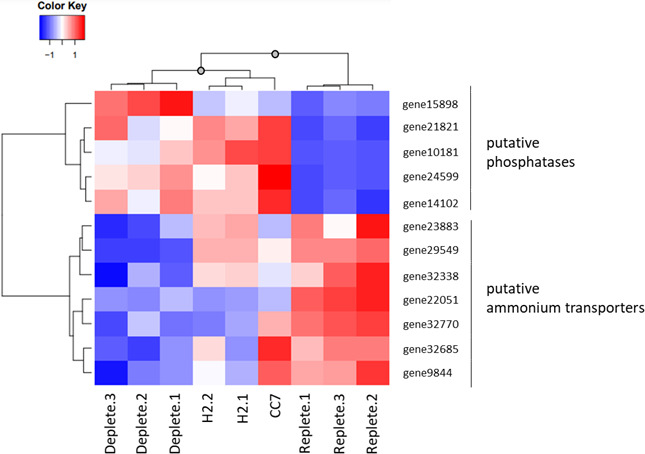
The expression pattern of putative phosphatases and ammonium transporters is similar between freshly isolated and phosphate-depleted *B. minutum* in culture. The 47 DEGs between replete and deplete cultures were functionally annotated using InterProScan, and GO enrichment analysis was performed using the GOEnrichment tool. Two molecular functions were overrepresented: acid phosphatase activity and ammonium transport activity. Normalized read counts of all putative phosphatases and ammonium transporters in the list of 47 DEGs were used to calculate Z-scores and to create a heatmap showing gene-to-gene Manhattan distance. Statistical significance of sample-to-sample clustering was assessed using the sigclust2 R package (30). Gray circles on the nodes indicate statistical significance (*P* < 5 × 10^−5^).

These 47 genes were functionally annotated using InterPro Scan and BLAST+. For 28 of them, a putative function was identifiable and GO terms were assigned (Table S1). GO enrichment analysis revealed several overrepresented molecular functions, including phosphatases and genes involved in N metabolism (Fig. S3; Table S2). Normalized read counts for phosphatases and ammonium transporters were used to calculate Z-scores and create a heatmap showing gene-to-gene distance (Fig. 4). Hierarchical clustering of gene-to-gene distance shows clear delineation between phosphatases and ammonium transporters. Generally, these genes have inverse expression profiles; while phosphatases are upregulated in deplete and freshly isolated samples, ammonium transporters are mostly downregulated. Because phosphatases play an important role in phosphate recycling by freeing P_i_ from phosphoesters, it makes sense that they are upregulated when phosphate is limited. Similarly, it is unsurprising that ammonium transporters are downregulated because there would be no need for symbionts to hoard N when phosphate is unavailable. Again, hierarchical clustering of sample-to-sample distance shows that the replete samples have a pattern of gene expression that is distinct from that of deplete and freshly isolated samples.

To further investigate genes involved in phosphate acquisition, conserved phosphate transporter domains were identified in the *B. minutum* genome using BLAST+ (RPS-BLAST). This yielded 32 putative phosphate transporter genes (Table S3). After combining RNAseq data from replete and deplete cultures, 18 of these genes were found to be differentially regulated in freshly isolated symbionts compared to those in culture (Data Set S4). Remarkably, 17 of the 18 DEGs were upregulated in freshly isolated samples, consistent with an organism that is phosphate limited and, thus, maximizing phosphate uptake. One gene was upregulated greater than ninefold in freshly isolated symbionts (gene7122). Interestingly, this gene contains an SPX domain; this domain is found in plant genes playing a variety of roles in responding to phosphate starvation (31).

**Fig 5:**
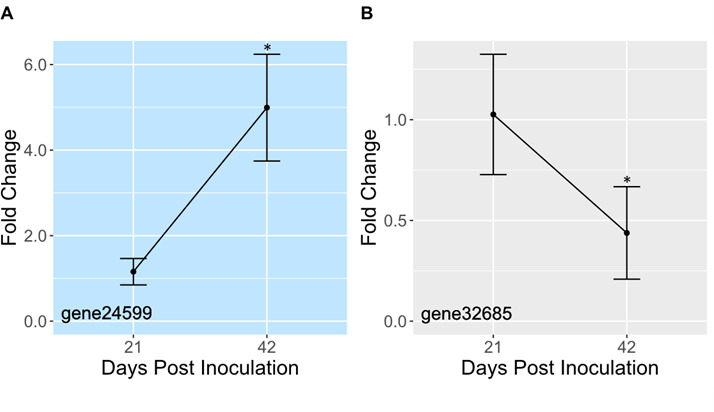
The expression of phosphatases and ammonium transporters changes with symbiosis establishment. Aposymbiotic CC7 anemones were inoculated with *B. minutum*. After 21 and 42 days, mRNA was isolated. Following reverse transcription, qPCR was run with gene-specific primers. Data were analyzed and plotted as the average fold change relative to day 21 ± SD (*n* = 3). * indicates that expression changes significantly between days 21 and 42 (Student’s *t*-test; *P* < 0.05). Light blue background indicates a phosphatase gene; gray indicates an ammonium transporter.

To determine how gene expression changes with symbiosis establishment, we measured expression of phosphatases and ammonium transporters 21 and 42 days after inoculating aposymbiotic CC7 anemones with *B. minutum*. Our prediction was that during establishment (day 21), algal division rates would be high and would slow once symbionts had fully populated the host (day 42). If control of algal proliferation was due to limited sharing of phosphate, then expression of phosphatases would be higher at day 42 than day 21. Likewise, ammonium transporters would be downregulated on day 42. Although algal density within the host increased ~2.5× (Fig. S4), only 2 of 12 phosphatases and ammonium transporters had significantly different expression levels from day 21 to day 42 (Fig. 5; Fig. S5). One phosphatase was upregulated by roughly fivefold (Fig. 5A), whereas an ammonium transporter was downregulated by approximately twofold (Fig. 5B). One possible explanation for the lack of significant results is that variability in symbiont density at day 21 was high; one anemone had nearly the same symbiont density at day 21 as the average symbiont density at day 42 (Fig. S4). Likewise, in our RNAseq analysis, patterns of phosphatase and ammonium transporter expression in freshly isolated symbionts were more variable than in cultures (Fig. 4).

### Phospholipids are replaced by neutral lipids in freshly isolated and phosphate-depleted *B. minutum* in culture

**Fig 6:**
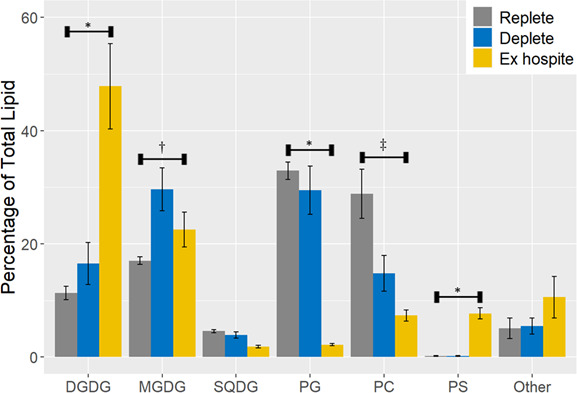
Phospholipids are replaced by neutral lipids in freshly isolated and phosphate-depleted *B. minutum* in culture. When the effect of phosphate deprivation on population growth became apparent on day 10, 2 × 10^7^ *B. minutum* cells were collected from each culture or from pooled *Exaiptasia*, flash frozen, and used for total lipid profiling with quadrupole tandem mass spectrometry. The mean contribution of each of the six most abundant lipids to the total lipidome is shown ± SD (*n* = 3). A two-way ANOVA with Bonferroni multiple comparisons test was used to determine significant differences between experimental groups. * indicates that freshly isolated symbionts are significantly different than replete and deplete, *P* < 0.05; † indicates that replete is significantly different than deplete, *P* < 0.05; ‡ indicates that all groups differ significantly from each other, *P* < 0.05. DGDG, digalactosyldiacylglycerol; MGDG, monogalatosyldiacylglycerol; SQDG, sulfoquinovosyldiacylglcerol; PG, phosphatidylglycerol; PC, phosphatidylcholine; PS, phosphatidylserine.

Not only is P_i_ lower in freshly isolated symbionts, but organic phosphate is as well. A major source of organic phosphate are phospholipids that are prevalent in all cellular membranes, thus, it is reasonable to assume that membrane composition would be sensitive to phosphate limitation. In fact, photosynthetic organisms, including Symbiodiniaceae, are known to substitute thylakoid phospholipids with the sulfolipid sulfoquinovosyldiacylglcerol (SQDG) to preserve photosynthetic efficiency when phosphate is limited (32, 33). Similarly, plasma membrane composition can change depending on phosphate availability (34, 35). To investigate this, we collected *B. minutum* from day 10 replete and deplete cultures, as well as from CC7-SSB01, and analyzed polar lipid composition by high resolution liquid chromatography/mass spectrometry. Predictably, when phosphate is limited (deplete), there is a significant reduction in the abundance of phospholipids, particularly phosphatidylcholine (PC), and a concomitant increase in the neutral lipid monogalactyldiacylglycerol (MGDG) (Fig. 6). Similarly, phospholipids (PC and phosphatidylglycerol [PG]) in freshly isolated symbionts appear to get replaced by neutral galactolipids (MGDG and digalactyldiacylglycerol [DGDG]).

## DISCUSSION

Consistent with other studies (19–21, 36), our experiments support the conclusion that *B. minutum* is deprived of phosphate when hosted by *E. diaphana*. Despite the host being fed *ad libitum* 3×/week, freshly isolated symbionts had no more phosphate per cell than phosphate-starved symbionts in culture (Fig. 2).

Further indicating that symbionts are phosphate-limited *in hospite*, RNAseq revealed that deplete and freshly isolated symbionts had similar patterns of gene expression for 47 phosphate-dependent genes (Fig. 3C and D), particularly phosphatases and ammonium transporters (Fig. 4). Phosphatases play an important role in phosphate recycling. Given that previous work has demonstrated phosphatase activity to be higher in freshly isolated symbionts than in cultures (19, 21), it is unsurprising that expression of phosphatases is elevated when phosphate is scarce. Indeed, two of the phosphatases identified in our screen are purple acid phosphatases, a highly conserved group that functions best in acidic environments. In cnidarians, endosymbionts reside within the symbiosome, a lysosome-derived organelle that maintains an acidic pH (37). Symbiont acid phosphatase activity has been pinpointed to the space between the symbiosome membrane and symbiont plasma membrane (19). A study of free-living Symbiodiniaceae investigated the use of different P sources (38). They concluded that although *Effrenium voratum* could use both organic and inorganic sources of P, organic phosphate was digested by alkaline phosphatases outside of the cell before free P_i_ was transported in. A similar mechanism may occur in *B. minutum in hospite*, except that instead of secreting alkaline phosphatases, they secrete acid phosphatases into the symbiosome. In keeping with a need to maximize phosphate uptake, 17 putative phosphate transporters were found to be upregulated in freshly isolated symbionts compared to those in culture (Table S3). Of particular interest are those genes identified as P_i_/H^+^ symporters because the symbiosome has a large H^+^ gradient with which to drive phosphate uptake.

It is worth noting that the activity of symbiont acid phosphatases can be inhibited in the presence of ~74 µM phosphate, whereas coral hosts are thought to maintain cytoplasmic phosphate concentrations of ~2 mM (19). If this is the case, then the relatively high activity of symbiont acid phosphatase in freshly isolated Symbiodiniaceae (19, 21) and *in hospite* (19) would indicate that the concentration of phosphate in the symbiosome is considerably lower than in host cytoplasm, suggesting that the host is actively limiting phosphate exchange. Further supporting micromolar concentrations of phosphate in the symbiosome is the low *K*_*M*_ of symbiont phosphate uptake (20). Although we do not know the concentration of P_i_ available in the cytoplasm of *Exaiptasia* stocks, it is not unreasonable to assume, given that they are fed to satiety 3×/week, that the number could be well above the 74 µM required to inhibit symbiont acid phosphatase. Future investigations should focus on clarifying this.

Interestingly, P_i_ accounted for a larger fraction of total phosphate in phosphate-limited cells (deplete and freshly isolated) than in replete cultures (~56% vs ~42%). This is consistent with an increase in phosphatase expression and, presumably, activity. By freeing P_i_ from phosphoesters, phosphatases can maintain a sufficient supply for making ATP and for phosphorylating enzymes to regulate metabolism and intracellular signaling. A major source of these phosphoesters would be the abundant phospholipids in cellular membranes. In fact, lipid profiling showed a marked decrease in phospholipid abundance in both deplete cultures and freshly isolated symbionts with a concomitant increase in the neutral lipids MGDG and DGDG (Fig. 6).

Under replete conditions, the membranes *of B. minutum* in culture are dominated by the phospholipids PG and PC, and the galactolipids MGDG and DGDG (Fig. 6). Most of these lipids belong to the thylakoid membrane; MGDG and DGDG are rarely found outside of the chloroplast, and PG is only a minor component of extraplastidial membranes (39). That such a large fraction of total lipid isolated from symbionts belongs to the thylakoid is plausible because the thylakoid constitutes a substantial portion of membrane surface area in any photosynthetic cell. When phosphate is scarce (deplete), there is a significant reduction in the abundance of phospholipids, particularly PC. Because PC is not found in the thylakoid (39), it can be assumed that this reflects a change in the plasma membrane. As for what replaces PC in the plasma membrane, the answer is not straightforward. In deplete cultures, there is a significant increase in MGDG, but this is a neutral lipid, not an anionic lipid like PC, and, as mentioned, it is generally relegated to the thylakoid. Strikingly, however, plants under phosphate deprivation can replace phospholipids in their plasma membranes with DGDG (40). Because MGDG is a precursor to DGDG, increased levels of MGDG seen in deplete samples may reflect increased production of DGDG. Indeed, although not significant, the percentage of DGDG present in deplete samples seems to be trending upward in comparison to replete. Consistent with phosphate deficiency *in hospite*, DGDG is, by far, the most abundant membrane lipid in freshly isolated samples, which corresponds with a significant reduction in PC compared to cultured symbionts. In contrast to PC, PS, which is undetectable in cultured symbionts, is abundant in symbionts *in hospite*. A literature review revealed no significant role for PS in the thylakoid, so we assume this is a change to plasma membrane composition, the consequences of which are unclear.

As for remodeling chloroplast membranes in response to phosphate limitation, there is no clear evidence in deplete samples. Perhaps if phosphate deprivation had lasted longer, we would see a clear reduction in the plastid phospholipid PG. We do, however, see a significant decrease in PG in freshly isolated samples, which may reflect that symbionts *in hospite* are even more phosphate limited than in deplete cultures. Interestingly, we could not confirm previous research indicating that P limitation induces a switch from anionic phospholipids to the anionic sulfolipid SQDG in the thylakoid to preserve phosphate availability (33). Instead, SQDG levels were the same in all samples. There are a few possible explanations for this discrepancy. It may reflect that our study employed *Breviolum*, whereas earlier work used a symbiont from the genus *Cladocopium*. Furthermore, earlier work investigated an imbalanced nutrient condition in which the concentration of N was high and phosphate ambient; in our study, the concentration of N on day 10 is unknown, but is certainly not high as the symbionts have sequestered it to generate nucleotides and amino acids necessary for population growth. In lieu of finding increased SQDG in freshly isolated samples, it seems likely that plastid PG, like PC in the plasma membrane, was replaced by DGDG. Further studies should investigate the presence of betaine lipids, which are structurally similar to phospholipids but lack P. The PC analog diacylglyceryl-N,N,N-trimethylhomoserine (DGTS) has been implicated in P-sparing in P-deprived *Chlorella kessleri* (41).

In addition to phosphatases, genes involved in N metabolism were significantly enriched in our RNAseq analysis. In deplete and freshly isolated samples, when phosphatase expression is upregulated, expression of several N metabolism genes, especially ammonium transporters, is significantly downregulated (Fig. 4). When symbionts have access to abundant N, they are limited by phosphate (42); thus, they may not sequester N when phosphate is unavailable. The consensus in the literature is that there is an ideal ratio of N to phosphate to maintain healthy symbiosis (43). In several species of coral, enrichment with P alone has no effect on algal proliferation; P, however, can work with N to enhance symbiont growth (14). This suggests that P would only limit symbiont growth *in hospite* if algae are kept in a N-enriched environment. Instead, substantial evidence indicates that N deprivation is used by cnidarians to limit symbiont proliferation (8, 10–13). In contrast to our results, a recent study showed that genes associated with N acquisition, including ammonium transporters, were upregulated *in hospite* and in N-deplete cultures compared to symbionts grown with sufficient N (13). Of 9 N acquisition genes increasing significantly from 16 to 40 days after reintroduction of symbionts in their study, only two were among the 47 phosphate-dependent genes we identified. One of these, gene23883 (identified as s6_38207 in [13]), had higher expression in freshly isolated symbionts than in deplete culture (Fig. 4), consistent with their findings. One confounding factor may be differences in our algal culture methods; their *B. minutum* were cultured in IMK media supplemented with casein hydrolysate. The N-deplete condition was IMK media without addition of casein hydrolysate. In our study, *B. minutum* was grown in f/2. IMK and f/2 differ in that f/2 has ~2.5× less N. Thus, our cultures had substantially less N to begin with. Nevertheless, future studies should carefully consider N and phosphate simultaneously. It is likely that cnidarians control the flow of both nutrients to regulate algal population growth.

**Fig 7:**
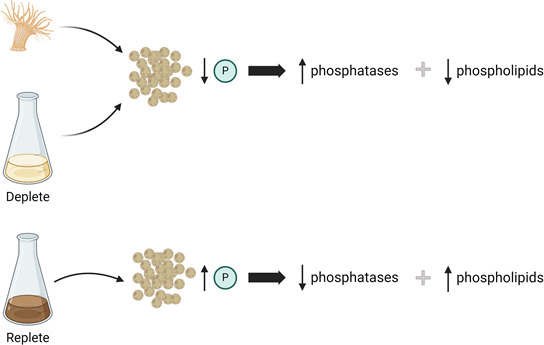
*B. minutum* is phosphate limited in the host as a means of limiting algal proliferation. Symbionts *in hospite* have lower levels of phosphate than algae cultured in phosphate-deficient conditions (Fig. 2). In response to phosphate deprivation, symbionts upregulate phosphatases (Fig. 4). These phosphatases then scavenge phosphates from phosphorylated macromolecules, particularly phospholipids, which are replaced by neutral lipids such as MGDG and DGDG (Fig. 6). As phosphate is required for algal proliferation (Fig. 1), a limited exchange of phosphate could be used by the host as a means of controlling algal division.

In conclusion, we have shown that symbionts *in hospite* are phosphate deficient. In response, they upregulate phosphatases to scavenge phosphates from phospholipids and potentially other phosphorylated macromolecules (Fig. 7). Several groups have proposed a model in which limited N sharing *in hospite* results from increased N acquisition by the host due to elevated production of algal photosynthetically fixed carbon (C) (10–13). In this model, when symbiont density is low, algae divide rapidly, but because of their limited population, the amount of photosynthate they share is low. Consequently, the host acquires ample N to encourage algal proliferation. The resulting increase in symbiont density increases sharing of photosynthate, causing the host to sequester more N for its own use and, thus, limit N availability in the symbiont. A similar self-regulating model could apply to phosphate as well. When algal densities are high *in hospite*, there is elevated sharing of photosynthate, which leads to the host keeping more phosphate to meet its own metabolic demands. The resulting phosphate limitation puts the brakes on algal proliferation. In this model, the connection between N/P metabolism and C accumulation is an essential component regulating the cnidarian-algal symbiosis. Consideration of this model may be important for understanding coral bleaching. Heat stress impairs photosynthesis (44), C fixation (45), and C translocation to the host (46). Under these conditions, the host’s demands for N and P would decrease, increasing availability to symbionts and causing an increase in algal proliferation similar to what has been reported (5). This decoupling of host-algal nutrient sharing has been observed in heat-stressed corals (47).

## MATERIALS AND METHODS

### Algal cultures

*B. minutum* was acquired from the National Center for Marine Algae (NCMA, Boothbay Harbor, ME, USA; accession CCMP3345). Cultures were grown in flasks containing silica-free f/2 (NCMA) made from filtered artificial seawater (ASW) in a growth chamber set to 27°C with a 13-h light period (~100 µmol*photons*m^2^*s^−1^). For phosphate limitation experiments, cells were seeded at 50,000 cells/mL in flasks containing standard silica-free f/2 (36.2 μM NaH_2_PO_4_; replete) or deplete f/2 (10 μM NaH_2_PO_4_). Phosphate concentrations in each culture were monitored using a colorimetric phosphate assay kit (Abcam, Cambridge, UK) following the manufacturer’s instructions. When the concentration of phosphate in deplete cultures reached the kit’s lower limit of detection (<1 µM), we began the experiment (day 1). Beginning on day 1, cultures were counted every 1–2 days by hemocytometer.

### Anemones

*E. diaphana* were maintained in ASW in a growth chamber set to 27°C with a 13-h light period (~40 µmol*photons*m^2^*s^−1^). Anemones were fed *Artemia* nauplii 3×/week and ASW was replaced immediately after feeding. Two clonal lines were used*—*H2-SSB01 and CC7-SSB01. The parental strains, H2 (26) and CC7 (27), had been made aposymbiotic using short-term cold shock (48) and reared long-term in the dark with periodic microscopic monitoring for the presence of symbionts. Prior to this study, H2-SSB01 and CC7-SSB01 were created from aposymbiotic anemones by adding *B. minutum* (SSB01) to the ASW at 50,000 cells/mL for 24 h before feeding. After feeding, ASW was replaced, and establishment of symbiosis was monitored. Symbiont density was assessed by removing tentacles and imaging chlorophyll autofluorescence at 40× magnification on an epifluorescence microscope using a Cy5 filter set. Average fluorescence in an area was determined following an established protocol (49). Stable symbiosis was maintained >6 mo before conducting experiments.

### Phosphate assay

To determine symbiont phosphate availability *in hospite*, one anemone (oral disk diameter ~0.5 mm) was homogenized using a Tissue-Tearor handheld homogenizer. Host tissue was removed by centrifuging at 4,700 × *g* for 3 min and washing 3× in ASW and 1× in 0.5% SDS in ASW. Pellets were resuspended in 0.5 mL of ASW and pipetted onto 0.5% Percoll (MilliporeSigma, Burlington, MA, USA). Purified algae were collected after centrifuging at 9,000 × *g* for 20 min. To determine phosphate availability in cultured symbionts, ~5 × 10^6^ cells were collected from replete or deplete cultures and washed 3× in ASW. Prior to the last wash, both freshly isolated and cultured symbionts were counted using a hemocytometer.

Algal pellets were dried overnight at 60°C, resuspended in 1 mL of 0.5 M NaOH with 25 mM Na_2_EDTA, and incubated for 16 h on a nutating mixer at room temperature. After centrifuging at 10,000 × *g* for 30 min, the supernatant was filtered through a 0.45-µm syringe filter and brought to 2 mL in ddH_2_O. Half the sample was taken to determine the concentration of P_i_; to the other half, 1 µL of 1 mg/mL sodium persulfate was added before autoclaving at 121°C for 30 min to free P_i_ from organic phosphate. This fraction was taken to determine the concentration of total phosphate. Phosphate concentrations were assessed using the same colorimetric phosphate assay kit used to monitor algal cultures.

### RNAseq

For RNA extraction, 2 × 10^7^ cells were collected from culture or by homogenizing 3–4 medium-sized *Exaiptasia* (oral disk diameter ~0.5 mm) using a Tissue-Tearor handheld homogenizer. Host tissue debris was removed by centrifuging at 5,000 × *g* for 3 min and washing 3× in ASW. Prior to proceeding, cells were flash frozen in liquid N_2_. RNA extraction combined lysis and homogenization in TRIzol (Life Technologies, Carlsbad, CA, USA) with a kit-based clean-up following a published protocol (50).

For sequencing, samples were transported frozen to the Clemson University Genomics and Bioinformatics Facility for processing (see the supplemental material for details). Demultiplexed reads were obtained from the sequencing facility, and analysis was performed on the opensource Galaxy Europe platform (usegalaxy.eu, v. 22.05 [51]) using a custom workflow (see Data Availability). Read counts were generated, and variance stabilization transformation (VST) (52) was applied using DESeq2. PCA and heatmaps were constructed in R (v. 4.2.1). Statistical significance of hierarchical clustering was determined using the sigclust2 R package (v. 1.2.4 [30]). Genes were functionally annotated using InterProScan and NCBI BLAST+. GO enrichment analysis was performed with GOEnrichment using Symbiodiniaceae GO annotations downloaded from reefgenomics.org.

For confirmation of the algal species used in our studies, the rRNA internal transcribed spacer 2 (ITS2) (53) consensus sequences were extracted from each transcriptome and aligned using MUSCLE (54). Sequences for cultured symbionts and those freshly isolated from anemones shared 100% identity with the *B. minutum* reference (Fig. S6). This variant is common among ≥3 species of *Breviolum* (55); however, based on the lineages of our stocks, we are confident that they are *B. minutum*.

### Quantitative RT-PCR

RNA (1 µg) was reverse transcribed using a QuantiTect Reverse Transcription Kit (Qiagen) following the manufacturer’s instructions. cDNA was diluted 1:5 in nuclease-free water before being used for qPCR. Reactions were set up as follows: 10 µL QuantiNova SYBR Green PCR Master Mix (Qiagen), 1.5 µL mixed forward and reverse primer (10 µM each, Table S4), 4.5 µL of nuclease-free water, and 4 µL of template. Reactions were run on a CFX96 (Bio-Rad, Hercules, CA, USA) at 95°C for 2 min for initial activation, followed by 40 cycles of 95°C for 5 s then 60°C for 10 s. Data were normalized to the expression of S-adenosyl methionine synthase (SAM) (56) and analyzed using the 2^−ΔΔCt^ method (57).

### Lipidomics

For lipid profiling, 2 × 10^7^ cells were collected from culture or by homogenizing 6–7 small *Exaiptasia* (oral disk diameter ~0.3 mm) using a Tissue-Tearor handheld homogenizer. Host tissue debris was removed as described above, and pellets were flash frozen in liquid N_2_, before being shipped overnight on dry ice to Creative Proteomics (Shirley, NY, USA) for polar lipid analysis (see the supplemental material for details).

## Data Availability

Raw RNAseq reads are available in NCBI’s Sequence Read Archive (Project PRJNA939342). DEG results (i.e., DESeq2) are included in the paper (Data Sets S1–S4). Our custom Galaxy Europe workflow is described in the supplemental material and can be accessed at https://usegalaxy.eu/u/mitchellgc/w/rnaseq-analysis.

## SUPPLEMENTAL MATERIAL

Data Set S1 mbio.01059-24-s0001.xlsx DESeq2 results for replete vs freshly isolated.

Data Set S2 mbio.01059-24-s0002.xlsx DESeq2 results for deplete vs freshly isolated.

Data Set S3 mbio.01059-24-s0003.xlsx DESeq2 results for replete vs deplete.

Data Set S4 mbio.01059-24-s0004.xlsx DESeq2 results for cultured vs freshly isolated.

Supplemental material mbio.01059-24-s0005.docx Supplemental text, figures, and tables.

ASM does not own the copyrights to Supplemental Material that may be linked to, or accessed through, an article. The authors have granted ASM a non-exclusive, world-wide license to publish the Supplemental Material files. Please contact the corresponding author directly for reuse.
