# Supplementary material for: Evidence for phosphate-dependent control of symbiont cell division in the model anemone Exaiptasia diaphana
Source: mBio. 2024 Aug 6;15(9):e01059-24. doi: 10.1128/mbio.01059-24 (PMC11389408; doi:10.1128/mbio.01059-24)
Supplement: Supplemental material — Supplemental text, figures, and tables. [file mbio.01059-24-s0005.docx]

**Supporting Information for**

Evidence for phosphate-dependent control of symbiont cell division in the model anemone *Exaiptasia diaphana*

Nathan G. Faulstich, Alexis R. Deloach, Ykok B. Ksor, Gabriel H. Mesa, Daiven S. Sharma, Sebastian L. Sisk, Geoffrey C. Mitchell

**This PDF file includes:**

Supporting Text

Figures S1 to S6

Tables S1 to S4

Legends for Datasets S1 to S4

Supporting Information Text

Materials and Methods

**RNA Extraction.** To control for gene expression differences due to mitotic phase, cultures were collected at the onset of the 13h light period, while most cells are in G0/G1 (1). Any effects of cell cycle phase on gene expression in hospite should be minimal since those symbionts divide infrequently (2). To extract RNA, pellets were resuspended in 1 mL of TRIzol, mixed thoroughly by pipetting, and transferred to screw-cap tubes with 0.3 g of glass beads (Sigma-Aldrich, St. Louis, MO). Cells were then disrupted twice for 90 sec in a MagNA Lyser (Roche Life Science, Basel, Switzerland) at 4,500 rpm. Debris was removed by centrifuging at 12,000 x g for 1 min, and supernatant was run through the RNeasy Plus Mini Kit following manufacturer’s instructions (Qiagen, Hilden, Germany). RNA was eluted in 30 μl of nuclease-free water and quantified on a NanoDrop Lite (Thermo Fisher Scientific, Waltham, MA).

**RNAseq:** Briefly, sample quality was assessed using a 2100 Bioanalyzer mRNA Nano assay (Agilent Technologies, Santa Clara, CA). Libraries were constructed from ~0.5-1 µg total RNA using the NEBNext Ultra II RNA Library Prep Kit for Illumina with the NEBNext Poly(A) mRNA magnetic isolation module (New England Biolabs, Ipswich, MA). Library quality was assessed using a 2100 Bioanalyzer high sensitivity DNA chip and quantified using KAPA qPCR (Roche, Basel, Switzerland). Libraries were loaded onto an Illumina NovaSeq 6000 SP flow cell for 300 cycles of 150 base-pair paired-end reads (1.5x10^7^ reads/sample).

**Galaxy Workflow.** Briefly, trimmomatic was run to remove low quality reads and exclude reads shorter than 50 bases. Trimmed reads were assessed with FastQC. Since freshly isolated samples were undoubtedly contaminated with host RNA, reads were mapped to a combined *Exaiptasia* (reefgenomics.org, v. 1.1) and *B. minutum* genome (https://doi.org/10.14264/uql.2019.745) using HISAT2. After removal of *Exaiptasia* reads, reads were counted using featureCounts and DESeq2 was run to find differentially expressed genes.

**Lipidomics.** Samples were resuspended in PBS before the addition of 12.5 mL of methanol and 6.25 mL of chloroform. Samples were shaken well, and 6.25 mL each of chloroform and water were added. After shaking again, samples were centrifuged at low speed for 10 min. to separate phases. The lower phase of each sample was collected. Another 6.25 mL of chloroform was added and this process was repeated. The collected lower phases were dried under N2 and resuspended in 0.5 mL of chloroform. Again, each sample was dried under N2 and then vacuum-dried overnight. Samples were weighed before being resuspended in 1 mL of chloroform and analyzed by ultra-performance liquid chromatography-tandem mass spectrometry (UPLC-MS/MS) on a Waters Xevo TQS (Milford, MA).

**Literature Cited**

1. Cato ML, Jester HD, Lavertu A, Lyman A, Tallent LM, Mitchell GC. 2019. Genome-wide analysis of cell cycle-regulating genes in the symbiotic dinoflagellate *Breviolum minutum*. G3-Genes Genom Genet 9:3843–3853.

2. Tivey TR, Parkinson JE, Weis VM. 2020. Host and symbiont cell cycle coordination is mediated by symbiotic state, nutrition, and partner identity in a model cnidarian-dinoflagellate symbiosis. mBio https://doi.org/10.1128/mbio.02626-19.


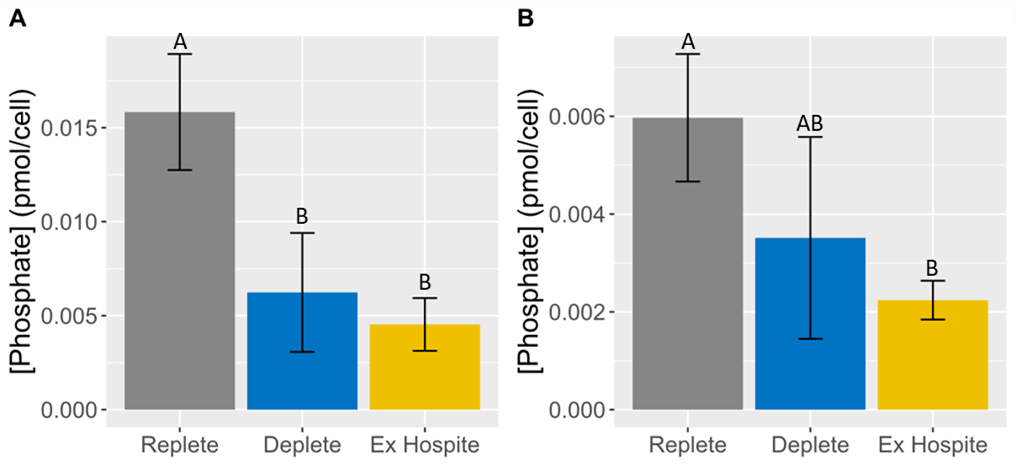


**Fig. S1. Available phosphate is as low in symbionts isolated from H2-SSB01 anemones as in phosphate-depleted *B. minutum* in culture.** When the effect of phosphate-deprivation on population growth became apparent on day 10, *B. minutum* were collected from replete and deplete cultures and H2-SSB01 anemones. The amount of total phosphate **(A)** and inorganic phosphate **(B)** per symbiont was determined using a colorimetric assay and plotted as the mean ± s.d. of 3 replicates. Groups that do not share a connecting letter are significantly different (1-way ANOVA followed by Tukey’s HSD, p < 0.005.


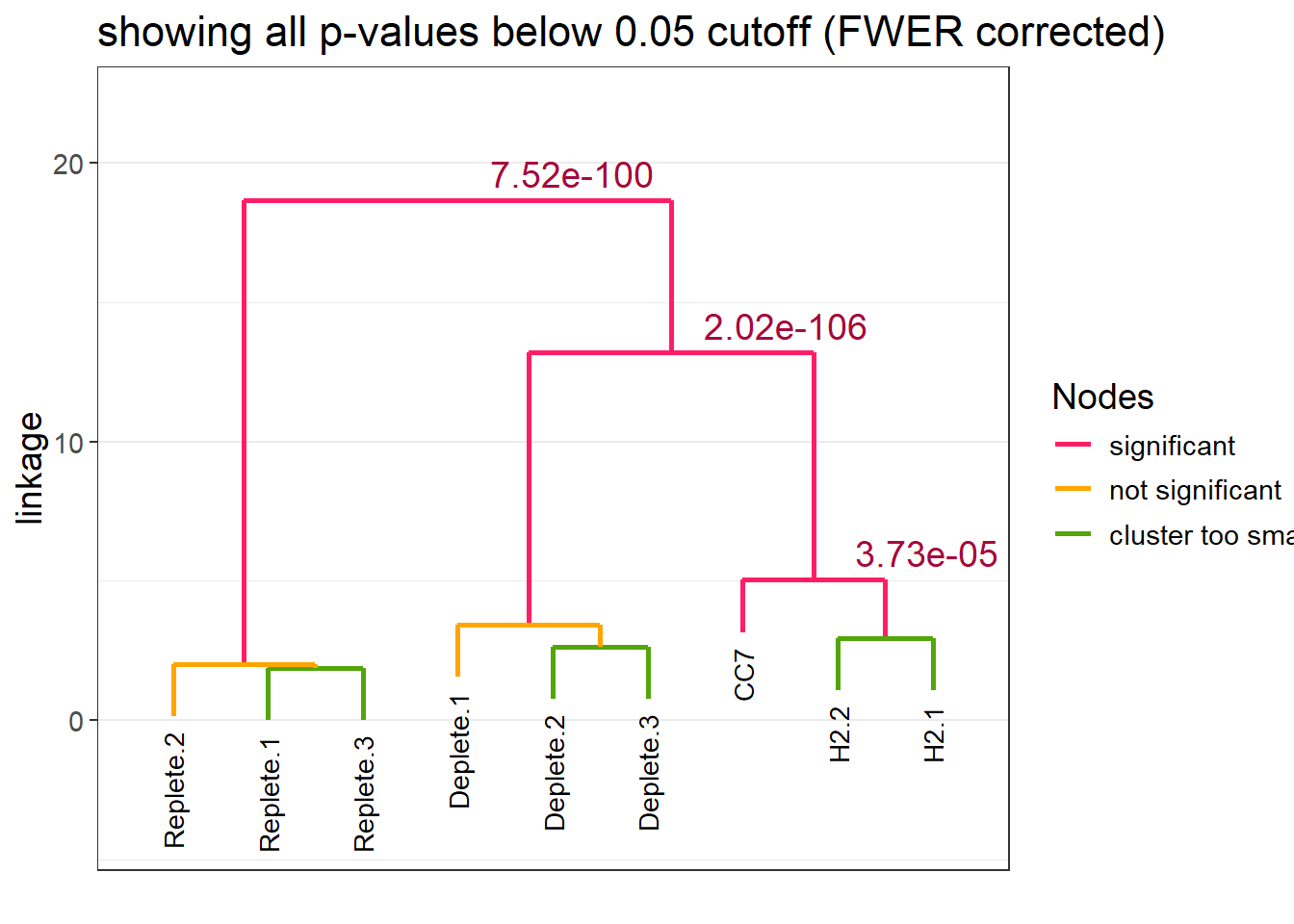


Fig. S2. The expression pattern of phosphorous-dependent genes is similar between freshly isolated and phosphorous-depleted *B. minutum* cultures. For RNAseq analysis, read counts were analyzed using DESeq2. VST-transformed expression data for these 47 DEG’s was used for hierarchical clustering of sample-to-sample Manhattan distance. Statistical significance of this clustering was assessed using the sigclust2 R package. Statistically significant nodes are shown in red with their corresponding p-values. Nodes in yellow are not statistically significant, while nodes in green lack the minimum number of samples (n ≥ 3) to assess statistical significance.


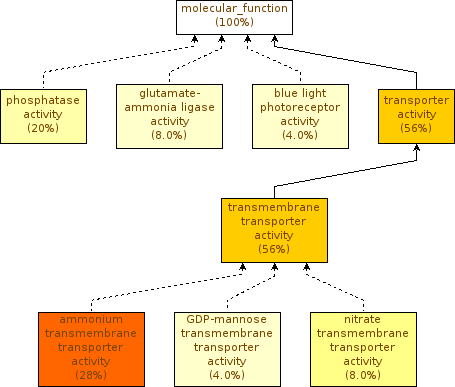


Fig. S3. Phosphatases and genes involved in nitrogen metabolism were overrepresented in the 47 phosphorus-dependent genes identified by RNAseq. The GOEnrichment tool was used to detect overrepresented GO terms in the 47 phosphorus-dependent genes identified by RNAseq. The graphical results for molecular function are shown.


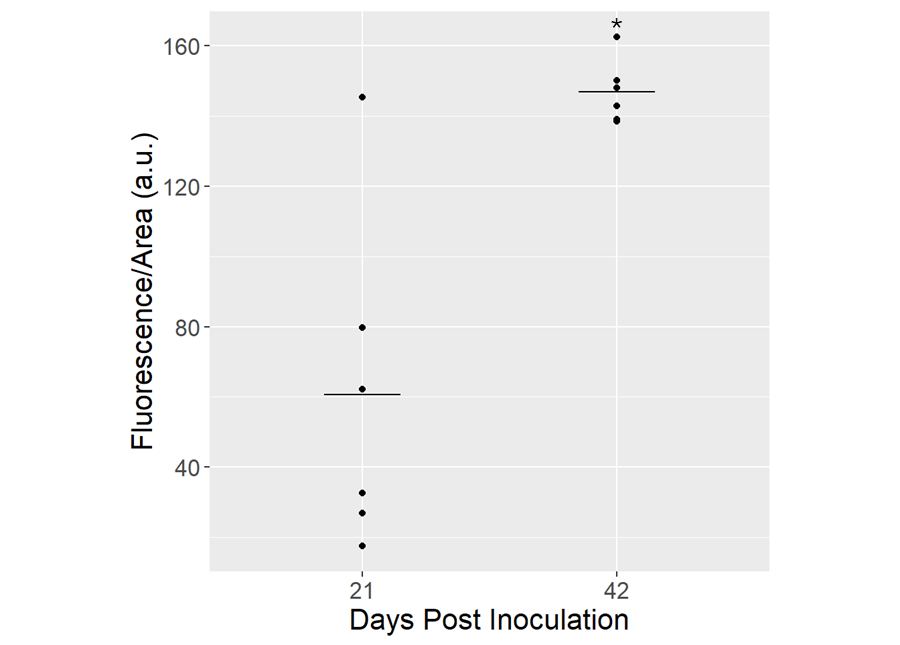


**Fig. S4. Symbiont density increases in CC7 anemones from 21 to 42 days post inoculation with B. *minutum*.** Aposymbiotic CC7 anemones were inoculated with *B. minutum*. After 21 and 42 days, tentacles were collected and chlorophyll autofluorescence was imaged on an epifluorescence microscope using a Cy5 filter set. Fluorescence per unit area was computed in Fiji and plotted per sample with a horizontal line showing the mean for each group (n = 5-6). (*) indicates that density changes significantly between days 21 and 42 (student’s t-test; p < 0.05).


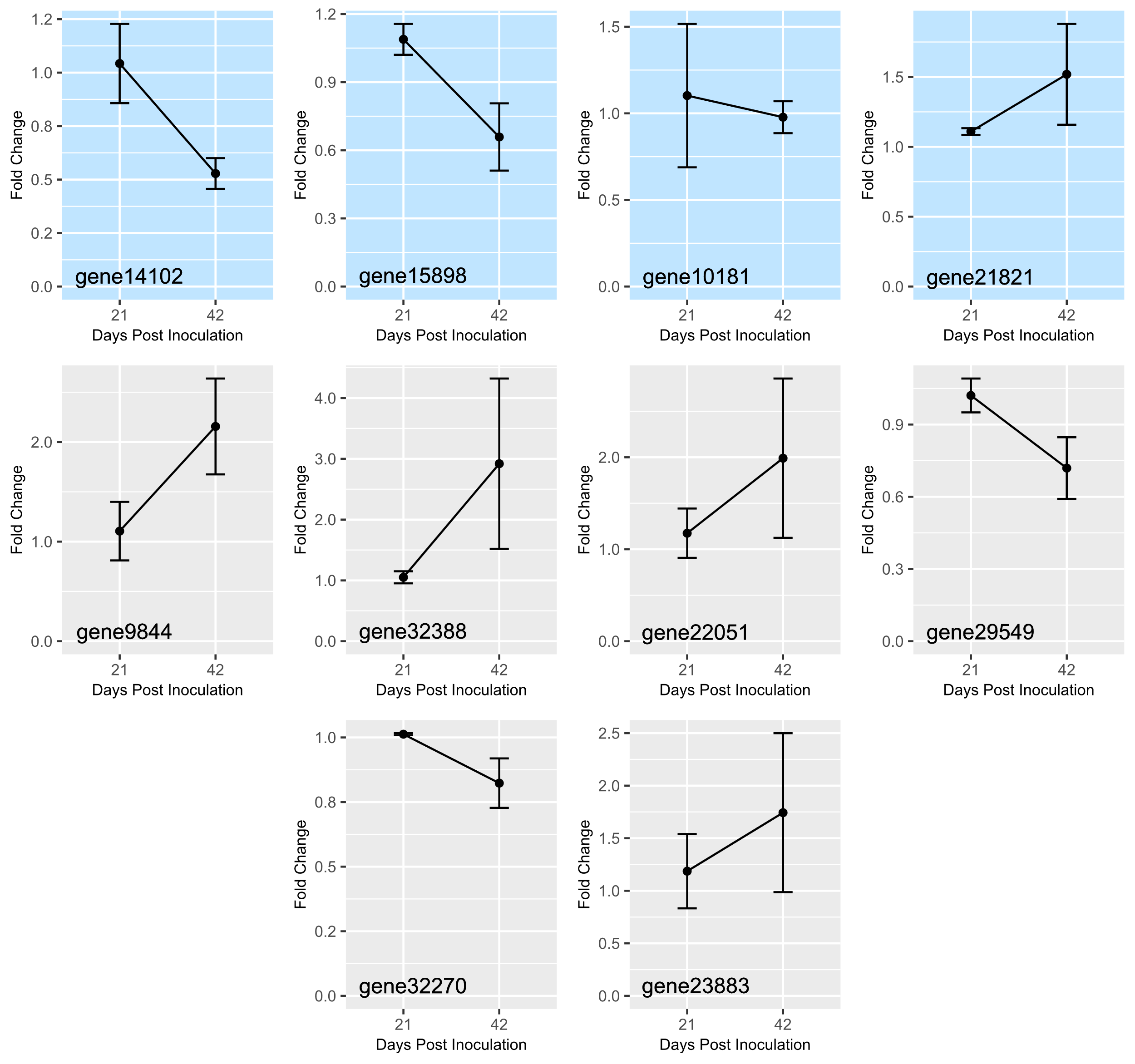


Fig. S5: Expression of phosphatases and ammonium transporters from 21 to 42 days post inoculation of aposymbiotic anemones with *B. minutum*. Aposymbiotic CC7 anemones were inoculated with *B. minutum*. After 21 and 42 days, mRNA was isolated. Following reverse transcription, qPCR was run with gene-specific primers. Data were analyzed and plotted as the average fold change relative to day 21 ± s.d. (n = 3). Light blue background indicates a phosphatase gene; grey indicates and ammonium transporter.


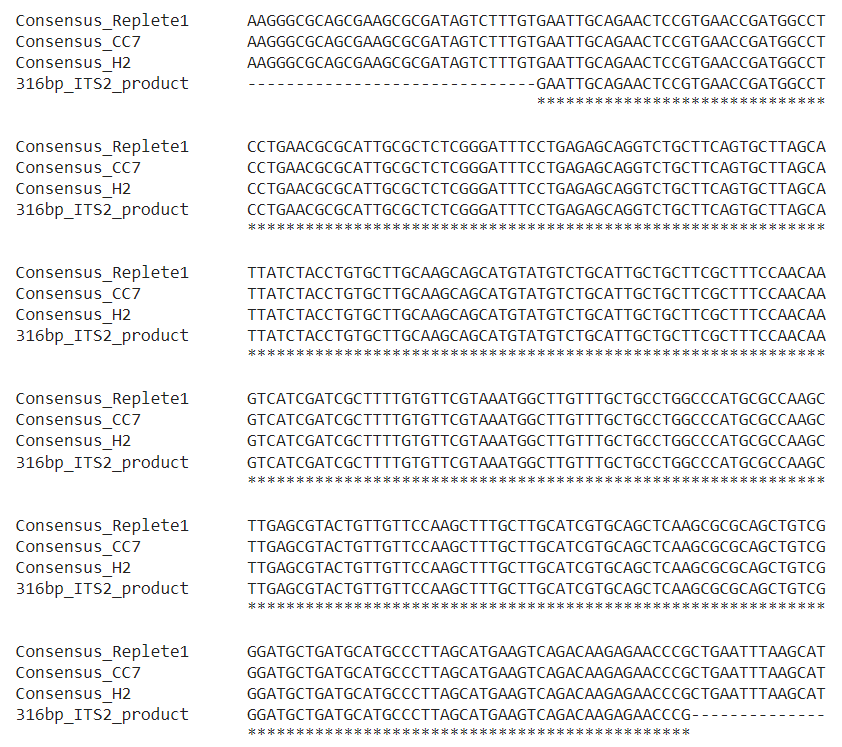


Fig. S6. Cultured and freshly isolated symbionts express identical ITS2 transcripts. As described in the main text, RNAseq reads were aligned to the *B. minutum* genome using HISAT2. The ivar consensus tool was used to determine the consensus sequence for the rRNA internal transcribed spacer (ITS2) for each sample. These sequences were aligned and compared to a *B. minutum* reference sequence using MUSCLE.

Table S1. GO terms associated with the 47 genes that are differentially expressed between replete and deplete samples. InterPro Scan and BLAST+ were used to annotate the 47 phosphorus-dependent genes identified by RNAseq. Gene functions and associated GO terms are shown.

| **GeneID** | **Putative Function** | **Relevant GO Terms** |
| --- | --- | --- |
| Bmin.gene32338.mRNA1 | ammonium transporter | GO:0008519\|GO:0016020\|GO:0072488 |
| Bmin.gene23883.mRNA1 | ammonium transporter | GO:0008519\|GO:0016020\|GO:0072488 |
| Bmin.gene22051.mRNA1 | ammonium transporter | GO:0008519\|GO:0016020\|GO:0072488 |
| Bmin.gene32685.mRNA1 | ammonium transporter | GO:0008519\|GO:0016020\|GO:0072488 |
| Bmin.gene32770.mRNA1 | ammonium transporter | GO:0008519\|GO:0016020\|GO:0072488 |
| Bmin.gene29549.mRNA1 | ammonium transporter | GO:0008519\|GO:0016020\|GO:0072488 |
| Bmin.gene9844.mRNA1 | ammonium transporter | GO:0008519\|GO:0016020\|GO:0072488 |
| Bmin.gene10181.mRNA1 | calcineurin-like phosphoesterase | GO:0016787 |
| Bmin.gene24599.mRNA1 | calcineurin-like phosphoesterase | GO:0016787 |
| Bmin.gene7797.mRNA1 | enolase | GO:0000015\|GO:0000287\|GO:0004634\|GO:0006096 |
| Bmin.gene17472.mRNA1 | GDP-mannose transporter | GO:0005458 |
| Bmin.gene471.mRNA1 | glutamine synthetase | GO:0004356\|GO:0006807 |
| Bmin.gene25401.mRNA1 | glutamine synthetase | GO:0004356\|GO:0006807 |
| Bmin.gene29597.mRNA1 | high-affinity nitrate transporter | GO:0015112 |
| Bmin.gene13574.mRNA1 | high-affinity nitrate transporter | GO:0015112 |
| Bmin.gene23581.mRNA1 | papain-like cysteine peptidase or xanthine/uracil permease | GO:0031177 |
| Bmin.gene2735.mRNA1 | ParB-like nuclease | GO:0007059 |
| Bmin.gene5977.mRNA1 | phototropin | GO:0009882 |
| Bmin.gene5801.mRNA1 | protein rolling stone | GO:0016020 |
| Bmin.gene21821.mRNA1 | purple acid phosphatase | GO:0003993\|GO:0046872\|GO:0016787 |
| Bmin.gene14102.mRNA1 | purple acid phosphatase | GO:0003993\|GO:0046872\|GO:0016787 |
| Bmin.gene15798.mRNA1 | regulator of chromosome condensation | GO:0007059 |
| Bmin.gene15898.mRNA1 | Ser/Thr protein phosphatase | GO:0004722 |
| Bmin.gene26111.mRNA1 | SAM synthase | GO:0004478\|GO:0006556 |
| Bmin.gene20797.mRNA1 | transmembrane amino acid transporter | GO:0015171 |
| Bmin.gene11945.mRNA1 | transmembrane amino acid transporter | GO:0015171 |
| Bmin.gene24962.mRNA1 | xanthine/uracil permease | GO:0015205 |
| Bmin.gene21561.mRNA1 | xanthine/uracil permease | GO:0015205 |
| Bmin.gene24389.mRNA1 | unknown |  |
| Bmin.gene26818.mRNA1 | unknown |  |
| Bmin.gene24388.mRNA1 | unknown |  |
| Bmin.gene10790.mRNA1 | unknown |  |
| Bmin.gene28881.mRNA1 | unknown |  |
| Bmin.gene21385.mRNA1 | unknown |  |
| Bmin.gene9959.mRNA1 | unknown |  |
| Bmin.gene22437.mRNA1 | unknown |  |
| Bmin.gene8611.mRNA1 | unknown |  |
| Bmin.gene8506.mRNA1 | unknown |  |
| Bmin.gene22867.mRNA1 | unknown |  |
| Bmin.gene15573.mRNA1 | unknown |  |
| Bmin.gene15574.mRNA1 | unknown |  |
| Bmin.gene2704.mRNA1 | unknown |  |
| Bmin.gene13944.mRNA1 | unknown |  |
| Bmin.gene7474.mRNA1 | unknown |  |
| Bmin.gene16033.mRNA1 | unknown |  |
| Bmin.gene23698.mRNA1 | unknown |  |
| Bmin.gene12295.mRNA1 | unknown |  |

**Table S2: Results of GO term enrichment analysis.** The GOEnrichment tool was used to detect overrepresented molecular function GO terms in the 47 phosphorus-dependent genes identified by RNAseq.

| **GO Term** | **Study #** | **Study Freq.** | **Pop. Freq.** | **p-value** | **q-value** | **Name** |
| --- | --- | --- | --- | --- | --- | --- |
| GO:0008519 | 7 | 28% | 0.24% | 8.97E-14 | 4.84E-12 | ammonium transmembrane transporter activity |
| GO:0022857 | 14 | 56% | 6.80% | 9.81E-11 | 2.65E-09 | transmembrane transporter activity |
| GO:0005215 | 14 | 56% | 7.00% | 1.43E-10 | 2.65E-09 | transporter activity |
| GO:0015112 | 2 | 8.00% | 0.02% | 3.45E-06 | 3.11E-05 | nitrate transmembrane transporter activity |
| GO:0016791 | 5 | 20% | 1.80% | 6.32E-05 | 2.44E-04 | phosphatase activity |
| GO:0004356 | 2 | 8.00% | 0.17% | 8.53E-04 | 2.88E-03 | glutamate-ammonia ligase activity |
| GO:0009882 | 1 | 4.00% | 0.01% | 1.90E-03 | 5.39E-03 | blue light photoreceptor activity |
| GO:0005458 | 1 | 4.00% | 0.01% | 1.90E-03 | 5.39E-03 | GDP-mannose transmembrane transporter activity |

Table S3. Several phosphate transporters are upregulated in freshly isolated symbionts compared to those in culture. Putative phosphate transporters in the *B. minutum* genome were identified by the presence of a conserved domain involved in phosphate transport (BLAST+, RPS-BLAST). A putative function and conserved domain (from either TIGRFAM or PFAM database) are shown. For RNAseq analysis, read counts for replete and deplete cultures were combined and compared to read counts from freshly isolated symbionts using DESeq2. Log_2_ fold-changes (cultured vs. freshly isolated) are shown, as are adjusted p-values. A dark line separates significant (p-adj. < 0.05) from non-significant results.

| **GeneID** | **log2(FC)** | **P-adj.** |  | **Putative Function** | **Conserved Domain** |
| --- | --- | --- | --- | --- | --- |
| Bmin.gene19158.mRNA1 | -0.769 | 8.07E-25 |  | triose phosphate transporter | TIGR00817 |
| Bmin.gene17032.mRNA1 | -0.762 | 2.74E-17 |  | triose phosphate transporter | TIGR00817 |
| Bmin.gene4515.mRNA1 | -0.854 | 6.75E-15 |  | Pi/H+ symporter | TIGR00887 |
| Bmin.gene21565.mRNA1 | -0.834 | 9.87E-13 |  | triose phosphate transporter | TIGR00817 |
| Bmin.gene7122.mRNA1 | -3.190 | 1.88E-12 |  | SPX domain-containing | pfam03105 |
| Bmin.gene27466.mRNA1 | -0.457 | 1.55E-11 |  | triose phosphate transporter | TIGR00817 |
| Bmin.gene832.mRNA1 | -0.644 | 1.97E-11 |  | triose phosphate transporter | TIGR00817 |
| Bmin.gene27275.mRNA1 | -0.866 | 7.12E-10 |  | Pi/H+ symporter | TIGR00887 |
| Bmin.gene15421.mRNA1 | -0.625 | 1.73E-06 |  | triose phosphate transporter | TIGR00817 |
| Bmin.gene3308.mRNA1 | -0.615 | 0.00015 |  | sodium-dependent Pi transporter | TIGR01013 |
| Bmin.gene1934.mRNA1 | -1.173 | 0.00019 |  | sodium-dependent Pi transporter | TIGR01013 |
| Bmin.gene4404.mRNA1 | -0.348 | 0.00033 |  | Pi/H+ symporter | TIGR00887 |
| Bmin.gene3103.mRNA1 | -0.402 | 0.00080 |  | sodium-dependent Pi transporter | TIGR01013 |
| Bmin.gene12828.mRNA1 | -0.375 | 0.00164 |  | triose phosphate transporter | TIGR00817 |
| Bmin.gene8529.mRNA1 | -0.315 | 0.00380 |  | triose phosphate transporter | TIGR00817 |
| Bmin.gene12274.mRNA1 | 0.263 | 0.00468 |  | Pi/H+ symporter | TIGR00887 |
| Bmin.gene22698.mRNA1 | -0.318 | 0.00605 |  | triose phosphate transporter | TIGR00817 |
| Bmin.gene22389.mRNA1 | -0.605 | 0.01402 |  | sodium-dependent Pi transporter | TIGR01013 |
| Bmin.gene18098.mRNA1 | -0.357 | 0.08748 |  | triose phosphate transporter | TIGR00817 |
| Bmin.gene18997.mRNA1 | 0.288 | 0.14562 |  | Pi/H+ symporter | TIGR00887 |
| Bmin.gene2951.mRNA1 | -0.342 | 0.15487 |  | triose phosphate transporter | TIGR00817 |
| Bmin.gene5005.mRNA1 | -0.178 | 0.20819 |  | triose phosphate transporter | TIGR00817 |
| Bmin.gene5474.mRNA1 | -0.160 | 0.25685 |  | triose phosphate transporter | TIGR00817 |
| Bmin.gene4039.mRNA1 | -0.155 | 0.32936 |  | phosphate:H+ symporter | TIGR00887 |
| Bmin.gene1400.mRNA1 | -0.100 | 0.40537 |  | triose phosphate transporter | TIGR00817 |
| Bmin.gene18267.mRNA1 | 0.087 | 0.52284 |  | triose phosphate transporter | TIGR00817 |
| Bmin.gene7394.mRNA1 | -0.081 | 0.53753 |  | Pi/H+ symporter | TIGR00887 |
| Bmin.gene8246.mRNA1 | -0.084 | 0.66347 |  | Pi/H+ symporter | TIGR00887 |
| Bmin.gene11151.mRNA1 | 0.061 | 0.78619 |  | triose phosphate transporter | TIGR00817 |
| Bmin.gene15188.mRNA1 | 0.061 | 0.78946 |  | triose phosphate transporter | TIGR00817 |
| Bmin.gene12733.mRNA1 | -0.028 | 0.89253 |  | triose phosphate transporter | TIGR00817 |
| Bmin.gene3362.mRNA1 | -0.037 | 0.91821 |  | SPX domain-containing | pfam03105 |

Table S4. Quantitative RT-PCR primers used to amplify phosphatase and ammonium transporter genes in *B. minutum*.

| **Gene Identifier** | **Forward Primer** | **Reverse Primer** |
| --- | --- | --- |
| gene9844 | CCAGTGAAACATCCTCCAGTT | TCATCACCTTGTCTGCCTTATC |
| gene10181 | GCAGATCCACGAGACTTTGT | AGCCAGAACCAGCCATATTC |
| gene14102 | CCAACCAACGCTATTGCTATTC | ATCAGGCCGACTTTGTATGG |
| gene15898 | CGGAGCATACTTATCCCAGTTC | ATCTCCTTGTGTGCGGTATTT |
| gene21821 | GCAGAGCGTTGGGATATCTT | CACCGTTATTCTGGGTCATCTC |
| gene22051 | CTTCGCCGGAATTGGATTCTA | CCATGACAACATGACGCTTTG |
| gene23883 | GGCAGGCTGAAGAAAGAGAA | CCGAGATACCAGTGAGACTTTG |
| gene24599 | TGCTCCTAACGGCATCTTTC | CTAGCTGTGTCCAACCAAACT |
| gene29549 | GTAGGCATGTGGAGAAGGAAG | CCCAACCAACCAAACCAAAG |
| gene32338 | TTGCAGGCTTGGTGTCTATC | ATGCACCCTGGTACACAATAC |
| gene32685 | GCAGACTTCAATCCTCAGAAGTA | CAAAGCCATGCAGAATCAAGAG |
| gene32770 | TGGCTTGGTCAGGTTCTTTC | CTCTTCCACATGGTCACTGTATC |

Dataset S1 (separate file). Results of differential gene expression analysis—replete vs. freshly isolated. For RNAseq analysis, read counts were analyzed using DESeq2. Raw results comparing replete cultures and freshly isolated symbionts are shown.

Dataset S2 (separate file). Results of differential gene expression analysis—deplete vs. freshly isolated. For RNAseq analysis, read counts were analyzed using DESeq2. Raw results comparing deplete cultures and freshly isolated symbionts are shown.

Dataset S3 (separate file). Results of differential gene expression analysis—replete vs. deplete. For RNAseq analysis, read counts were analyzed using DESeq2. Raw results comparing replete and deplete cultures are shown.

Dataset S4 (separate file). Results of differential gene expression analysis—cultured vs. freshly isolated. For RNAseq analysis, read counts were analyzed using DESeq2. Raw results comparing cultured and freshly isolated symbionts are shown.
